# PractiCPP: a deep learning approach tailored for extremely imbalanced datasets in cell-penetrating peptide prediction

**DOI:** 10.1093/bioinformatics/btae058

**Published:** 2024-02-01

**Authors:** Kexin Shi, Yuanpeng Xiong, Yu Wang, Yifan Deng, Wenjia Wang, Bingyi Jing, Xin Gao

**Affiliations:** Syneron Technology, Guangzhou 510000, China; Individualized Interdisciplinary Program (Data Science and Analytics), The Hong Kong University of Science and Technology, Hong Kong SAR, China; Syneron Technology, Guangzhou 510000, China; Syneron Technology, Guangzhou 510000, China; Syneron Technology, Guangzhou 510000, China; Data Science and Analytics Thrust, The Hong Kong University of Science and Technology (Guangzhou), Nansha, Guangzhou, 511400, Guangdong, China; Department of Statistics and Data Science, Southern University of Science and Technology, Shenzhen 518000, China; Syneron Technology, Guangzhou 510000, China; Computer Science Program, Computer, Electrical and Mathematical Sciences and Engineering Division, King Abdullah University of Science and Technology (KAUST), Thuwal 23955, Saudi Arabia; Computational Bioscience Research Center, King Abdullah University of Science and Technology (KAUST), Thuwal 23955, Saudi Arabia

## Abstract

**Motivation:**

Effective drug delivery systems are paramount in enhancing pharmaceutical outcomes, particularly through the use of cell-penetrating peptides (CPPs). These peptides are gaining prominence due to their ability to penetrate eukaryotic cells efficiently without inflicting significant damage to the cellular membrane, thereby ensuring optimal drug delivery. However, the identification and characterization of CPPs remain a challenge due to the laborious and time-consuming nature of conventional methods, despite advances in proteomics. Current computational models, however, are predominantly tailored for balanced datasets, an approach that falls short in real-world applications characterized by a scarcity of known positive CPP instances.

**Results:**

To navigate this shortfall, we introduce PractiCPP, a novel deep-learning framework tailored for CPP prediction in highly imbalanced data scenarios. Uniquely designed with the integration of hard negative sampling and a sophisticated feature extraction and prediction module, PractiCPP facilitates an intricate understanding and learning from imbalanced data. Our extensive computational validations highlight PractiCPP’s exceptional ability to outperform existing state-of-the-art methods, demonstrating remarkable accuracy, even in datasets with an extreme positive-to-negative ratio of 1:1000. Furthermore, through methodical embedding visualizations, we have established that models trained on balanced datasets are not conducive to practical, large-scale CPP identification, as they do not accurately reflect real-world complexities. In summary, PractiCPP potentially offers new perspectives in CPP prediction methodologies. Its design and validation, informed by real-world dataset constraints, suggest its utility as a valuable tool in supporting the acceleration of drug delivery advancements.

**Availability and implementation:**

The source code of PractiCPP is available on Figshare at https://doi.org/10.6084/m9.figshare.25053878.v1.

## 1 Introduction

In the realm of therapeutic treatment, the efficiency of drug delivery signs significantly impacts the therapeutic efficacy of pharmaceuticals (Zhang *et al.* 2013). To enhance drug delivery efficiency and augment the interventional impact on intracellular targets, researchers have developed various drug delivery systems, including cell-penetrating peptides (CPPs) (Qian *et al.* 2014). CPPs, typically consisting of 5–30 amino acids, possess the ability to penetrate eukaryotic cells without causing substantial damage to the cell membrane (Qian *et al.* 2014). Consequently, CPPs hold potential for delivering membrane-impermeable cargoes, such as peptides, proteins, nucleic acids, and nanoparticles, into the interior of mammalian cells as novel therapeutics. Since the initial discovery of Tat (Richard *et al.* 2003) (Truncated HIV-1 Tat protein basic domain, which rapidly translocates through the plasma membrane and accumulates in the cell nucleus), thousands of CPPs have been reported (Gautam *et al.* 2012). These CPPs are categorized into three types based on their topological structures: linear peptides, cyclic peptides, and bicyclic peptides. Peptides with different topological structures exhibit varying loading capacities, thereby influencing the efficacy of the final drug delivery (Buyanova *et al.* 2022).

The advent of next-generation proteomics technologies has facilitated the sequencing of peptide and protein molecules (Altelaar *et al.* 2013). Pei *et al.* used gene editing technology and phage display technology to design novel polypeptides (Rhodes and Pei 2017) and utilized fluorescent labeling and Caco-2 cell array technology to assess the membrane penetration ability of the polypeptides (Dougherty *et al.* 2019). However, the identification and characterization of specific CPPs with optimal uptake efficiency through conventional in vitro assays remain time-consuming and labor-intensive (Ragin *et al.* 2002). On the other hand, the membrane permeability and cellular uptake efficiency of penetrating peptides are closely related to their sequence characteristics (Schmidt *et al.* 2010) and physical and chemical properties (Milletti 2012). Therefore, the development of computational methods is a rational choice for accurately identifying prospective CPPs, thereby reducing the experimental burden on researchers (Manavalan and Patra 2022).

Recently, machine learning has been successfully applied to numerous computational biology and chemistry problems, including drug–target interaction prediction (Chen *et al.* 2016), blood–brain barrier permeability prediction (Liu *et al.* 2004), and passive membrane permeability prediction (Lomize *et al.* 2019). Several statistical methods have also been proposed for CPP prediction, such as ARF motif based preditor (Johansson *et al.* 2008). Subsequently, a multitude of algorithms based on statistical learning and deep learning have been continuously proposed, including CPPpred (Holton *et al.* 2013), CellPPD (Gautam *et al.* 2015), C2Pred (Tang *et al.* 2016), SkipCPP-Pred (Wei *et al.* 2017a), CPPred-RF (Wei *et al.* 2017b), MLCPP-2.0 (Manavalan and Patra 2022), and BChemRF-CPPred (de Oliveira *et al.* 2021). Recent studies have provided a detailed description of existing CPP prediction methods in terms of algorithms, feature encodings, and evaluation strategies (Su *et al.* 2020). Some of these methods cannot only predict whether molecules can pass through the cell membrane but also predict their endocytosis efficiency, such as CPPred-RF, StackCPPred, and MLCPP-2.0. However, existing methods often focus on balanced datasets, which is fundamentally different from real-world scenarios where only a limited number of known positive CPP samples are available, while a large number of peptides have an undetermined CPP status.

In this study, we propose a deep learning framework, PractiCPP, specifically designed for the extremely imbalanced binary classification challenge inherent to CPP prediction. PractiCPP primarily consists of two components: (i) hard negative sampling, which selectively incorporates challenging negative samples from the negative set, and (ii) feature extraction and prediction module, termed as PractiCPPbase, which extracts three distinct features: sequential features, local structure features, and pretrained features originated from large language model (LLM). This approach not only mitigates the imbalance between negative and positive instances but also forces the model to learn more fine-grained features, consequently enhancing its overall performance.

Comprehensive experiments demonstrate that PractiCPP exhibits outstanding prediction performance on both balanced and imbalanced datasets. In the balanced dataset (CPP924), PractiCPP consistently outperforms seven top-performing baselines in terms of accuracy, sensitivity, specificity, and MCC. On the imbalanced dataset with a positive-to-negative ratio of 1:1000, PractiCPP yields the best performance among all baselines under various evaluation metrics, such as AUPR (the area under the precision–recall curve), precision, F1 score, and FP per correct (the average number of false positive samples for each correctly predicted sample). We also visualize the embeddings generated by different versions of our model and exhibit that our models trained on balanced data might be impractical for guiding large-scale peptide selection for wet-lab experiments in CPP identification while training with imbalanced data has the potential to empower real-world applications. In summary, PractiCPP offers a promising solution for the prediction of CPPs in real-world scenarios, where only a limited number of known positive CPP samples are available. The model’s ability to handle imbalanced data and its state-of-the-art performance on both balanced and imbalanced datasets demonstrate its potential for practical deployment in drug delivery research and development.

## 2 Materials and methods

### 2.1 Datasets

In this paper, we use two distinct datasets for training and testing our model, i.e. balanced dataset CPP924 and 1:1000 imbalanced dataset. The CPP924 dataset is obtained from CPPsite 2.0 (Kardani and Bolhassani 2021), containing 462 known CPPs and 462 non-CPPs, which is commonly used in CPP prediction tasks. For a fair comparison, we also evaluate the proposed method PractiCPP on this balanced CPP924 dataset.

To form a 1:1000 imbalanced dataset, the positive samples primarily originated from CPP924 and CPPsite3 in StackCPPred (Fu *et al.* 2020). To avoid sequence redundancy, we use CD-HIT (Fu *et al.* 2012) for sequence deduplication, with a threshold of 80%. Additionally, we remove data containing non-natural amino acids, as the modification of non-natural amino acids in peptides is a more general topic beyond the scope of this paper. Furthermore, we eliminate some sequences with conflicting labels based on cross-comparison. These sequences are marked as highly membrane-permeable in some studies, while in others, they are found to have weak or non-permeable properties (Gautam *et al.* 2012). Ultimately, it results in a balanced dataset consisting of 649 positive samples. To construct the negative set, we download 17 059 888 protein sequences from UniProt (Consortium 2019) and PeptideAtlas (Deutsch *et al.* 2008) and filter the data with a length threshold of 50, the maximum size of peptide based on its definition. To prevent data leakage, we also perform deduplication using CD-HIT at an 80% threshold (both individually and in combination with the positive samples), resulting in a final dataset of 16 689 857 sequences. These sequences are generally considered negative samples, but in reality, they should be regarded as an unlabeled dataset. Then, we randomly sample 649 000 samples from this unlabeled dataset to form a 1:1000 imbalanced dataset. In addition, we split out an independent test set where the ratio of positive to negative samples is maintained at 1:1000 for performance evaluation.

### 2.2 Framework of PractiCPP

Previous studies on CPP prediction typically focus on balanced datasets with a 1:1 ratio of positive to negative samples. However, our work adopts a more practical approach. In real-world contexts, we only have a limited number of laboratory-verified CPPs, and there is an overwhelming amount of unlabeled peptides whose actual status (CPP or non-CPP) remains unknown. To streamline our model representation, we approach this scenario as an imbalanced two-class classification problem, where the ratio between positive samples and negative samples is approximately 1:1000. Here, we refer to unlabeled data as the negative data.

**Figure 1.**
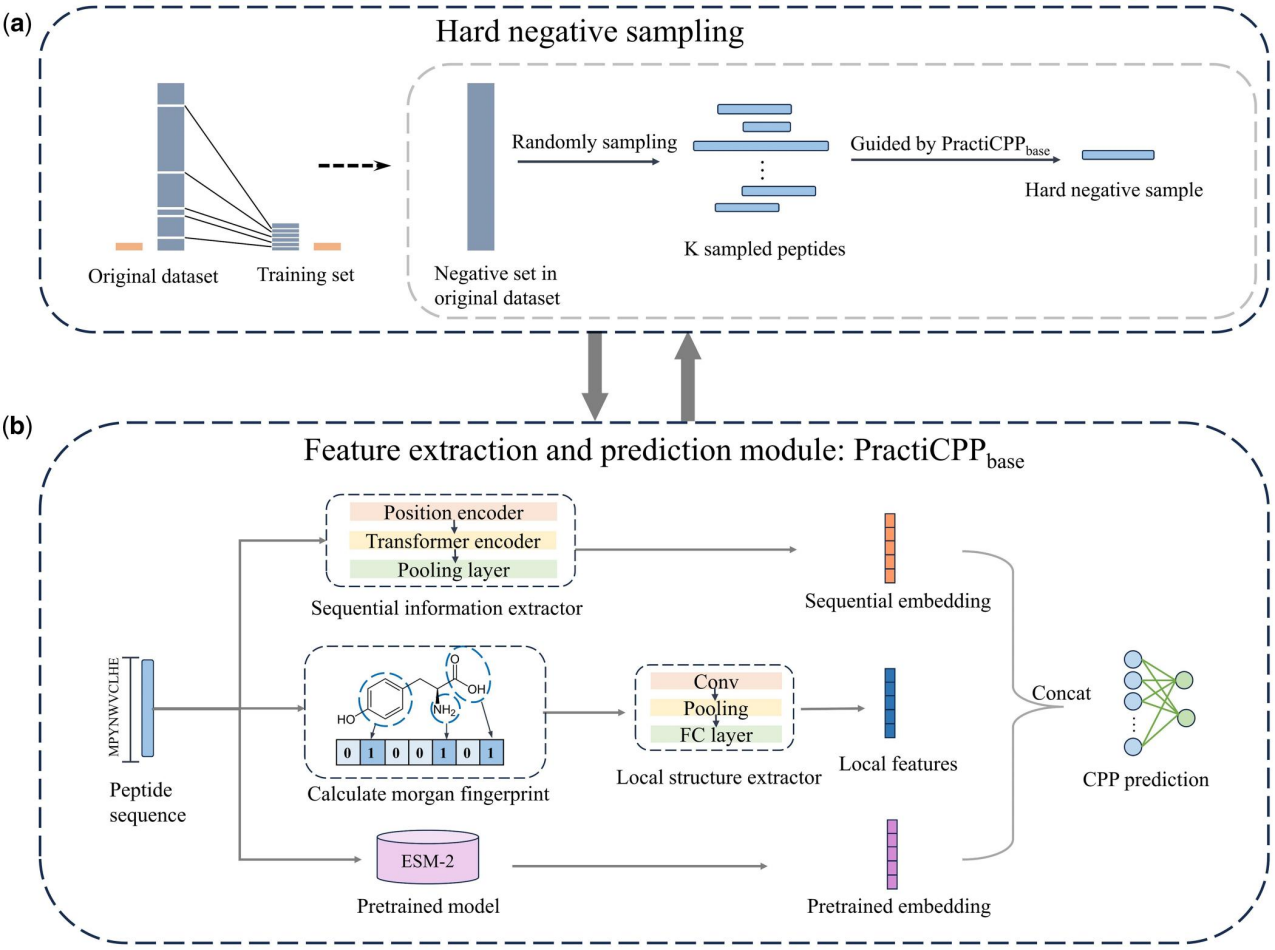
Workflow of PractiCPP for imbalanced CPP prediction. It includes two components: (a) hard negative sampling to select the most challenging negative instances for model updating. (b) Feature extraction and prediction module (named PractiCPP${\;}_{\text{base}}$) that aims at extracting three types of peptide features: sequential features from peptide sequences, local features from peptides’ Morgan fingerprints and pretrained features. The hard negative sampling process is guided by PractiCPP${\;}_{\text{base}}$, and these two steps are executed iteratively until model convergence.

In this study, we propose a deep learning framework, PractiCPP, designed specifically for the imbalanced binary classification challenge inherent to CPP prediction. PractiCPP mainly consists of two components: (i) hard negative sampling, and (ii) feature extraction and prediction module, termed as PractiCPP${\;}_{\text{base}}$, constituting the base model of PractiCPP. These two steps are repeated iteratively until convergence (Fig. 1).

During the hard negative sampling phase, we selectively incorporate challenging negative samples from the negative set, pairing them with positive instances for model training.

In the feature extraction and prediction module, we use solely the peptide sequence (amino acid sequence) as our input. From this, we derive three distinct features: sequential features, local structure features and pretrained features, using varied techniques.

#### 2.2.1 Hard negative sampling

Hard negative sampling (Rendle and Freudenthaler 2014) is adopted to address the challenges presented by the severe class imbalance in our dataset, by selecting negative examples that the model finds most challenging to update the model, thereby refining its decision boundaries for improved generalization.

Specifically, let P be the positive set, N the negative set and $P_{\text{batch}}$ the positive instances in a batch. Given a batch containing positive instances, we first randomly sample a subset of negative instances $N_{\text{sample}}$ with a size of $K\times |P_{\text{batch}}|$ from the whole negative set N. It can be represented as follows:

$$N_{\text{sample}}\in N\;\text{and}\;|N_{\text{sample}}|=K\times |P_{\text{batch}}|,$$

where $\left|P_{\text{batch}}\right|$ denotes the number of positive instances in the current batch and *K* (with K≥1) is a manually defined multiplicative factor.

Next, we apply PractiCPP${\;}_{\text{base}}$ to compute the probability of instances in $N_{\text{sample}}$ being classified as positive. We then select the top $M\times |P_{\text{batch}}|(1\leq M\leq K)$ negatives with the highest probabilities, denoted as $N_{\text{hard}}$. To balance the computational efficiency with the adequate representation of the negative set in our imbalanced classification task, we empirically set the negative sampling ratio *M* to 3 as suggested in previous works (Yang *et al.* 2016). In general, the selection of $N_{\text{hard}}$ can be represented as follows:

$$N_{\text{hard}}={\text{TOP}}_{3\times |P_{\text{batch}}|}({\text{PractiCPP}}_{\text{base}}(N_{\text{sample}})).$$

Then, the training set T for the current batch is constituted by merging $N_{\text{hard}}$ with $P_{\text{batch}}$ as follows:

$$T=P_{\text{batch}}\cup N_{\text{hard}}.$$

Note that the choice of *K* plays a critical role in regulating the difficulty of the negatives in the training set. Specifically, a larger *K* broadens the sampled subset $N_{\text{sample}}$, increasing the likelihood of capturing negatives that are closer to the classifier’s decision boundary, thus increasing the hardness level of $N_{\text{hard}}$. In experiments on imbalanced data, we search *K* in {3,9,15,21,30} and the optimal performance is observed at K=9. (See Supplementary Notes S2 for a detailed analysis.)

#### 2.2.2 Sequential feature extraction in PractiCPP${\;}_{\text{base}}$

For a peptide $p=(a_{1},a_{2},\ldots ,a_{l})$, where $a_{i}$ is the amino acid at position *i* and *l* is the sequence length, we map it to a numeric vector $v_{p}$ by uniquely numbering each amino acid and padding to a consistent length. In addition, we generate the positional embedding for $v_{p}$ to encode positional information of each amino acid in *p* as follows:

$$x=v_{p}+\text{Pos}(v_{p}),$$

where Pos(⋅) is the positional information encoder utilizing sine and cosine functions (Vaswani *et al.* 2017), and *x*, which combines $v_{p}$ and its positional embedding, act as the input to a transformer encoder layer. Transformer encoding operation (Vaswani *et al.* 2017) is as follows:

$$\begin{array}{c} x^{\prime }=\text{LayerNorm}(x+\text{MultiHead}(x)),\\ x_{\text{Trans}}=\text{LayerNorm}(x^{\prime }+\text{FeedForward}(x^{\prime })), \end{array}$$

where $x_{\text{Trans}}$ represents the embedding derived from the transformer encoder, MultiHead(|⋅|) denotes the multi-head attention mechanism, LayerNorm(⋅) is the layer normalization, and FeedForward is a feed-forward network. Then, we apply a pooling layer Pool(⋅), which aggregates the information from the encoder’s output to generate the sequential features $x_{\text{seq}}\in R^{1\times 512}$ of a peptide, as follows:

$$x_{\text{seq}}=\text{Pool}(x_{\text{Trans}})$$

#### 2.2.3 Local feature extraction in PractiCPP${\;}_{\text{base}}$

The Morgan fingerprint (Rogers and Hahn 2010) is a molecular descriptor used in cheminformatics to capture the local structural environment of each atom in a molecule. Peptides are essentially small to medium-sized polymers and have distinct atoms, bonds, and functional groups like any other organic molecule. Thus, we treat peptides as molecules and compute their Morgan fingerprints to get the structural features of peptides.

Specifically, let *O* be the set of atoms in peptide *p* and $L_{n}(o)$ denote the label of atom o∈O after *n* iterations, then:

$$\begin{array}{l} L_{0}(o)=\text{Init}(o),\\ L_{n+1}(o)=\text{Hash}\left(L_{n}(o)\cup \underset{b\in \text{Neighbors}(o)}{\cup }L_{n}(b)\right), \end{array}$$

where Init(o) is the initial label of atom *o*, Hash(⋅) is the hashing function used to generate a new label, and Neighbors(o) is the set of atoms bonded to atom *o*. this process is repeated until a specified radius from each atom is reached (we set the radius as 2 in experiments). Finally, the generated labels from each iteration, which represent atom local environments, are hashed into a bit vector *fingerprint* of length 1024 as follows:

$$\mathrm{fingerprint}=(b_{1},b_{2},\ldots ,b_{1024}),$$

where $b_{i}\in \left\{0,1\right\}\;\text{for}\;i=1,2,\ldots ,1024.$ To fully exploit local structure patterns in the fingerprint, we use a 1D convolution layer to detect adjacent bit interactions as follows:

$$x_{\text{local}}=\text{FC}(\text{Pool}(\text{Conv}(\mathrm{fingerprint}))),$$

where $x_{\text{local}}\in R^{1\times 512}$ is generated local features of a peptide, with Conv(⋅) representing a 1-d convolution layer, Pool(⋅) the max-pooling operation and FC(⋅) a fully connected layer.

#### 2.2.4 Pretrained features in PractiCPP${\;}_{\text{base}}$

In this study, the available dataset for model training is restricted to a few hundred positive samples, specifically cell-penetrating peptides. Such a limitation often hampers the accuracy of feature extraction (Thirunavukarasu *et al.* 2023). Recently, advanced language models developed for protein structures have emerged (Brandes *et al.* 2023). These models, trained on larger datasets, can transfer knowledge, improving performance on limited datasets and enhancing our understanding of peptide attributes. In this context, we use ESM-2 (Lin *et al.* 2022), a cutting-edge language model for large-scale protein structure prediction, to generate pretrained feature embeddings $x_{\text{pre}}$ in our experiments.

Specifically, for a peptide, we first tokenize its sequence into individual amino acids and map them to a numeric vector $v_{p}\in R^{1\times l}$ with one-hot encoding, where *l* is the sequence length. Then, for *i*th position in the sequence, a contextualized representation $H_{i}\in R^{1\times 1280}$ is computed with the pretrained ESM-2 model. Hence, we have:

$$H=\text{ESM}-2(v_{p}),$$

where $H\in R^{l\times 1280}$. Next, we apply a mean pooling operation on the contextualized representations *H* to obtain a single embedding vector $x_{\text{esm}}\in R^{1\times 1280}$:

$$x_{\text{esm}}=\left(\sum_{i=1}^{l}H_{i}\right)/l.$$

Finally, a fully connected layer FC(⋅) is used to generate the pretrained embedding $x_{\text{pre}}\in R^{1\times 512}$:

$$x_{\text{pre}}=\text{FC}(x_{\text{esm}})$$

To form a comprehensive representation of each peptide, the above three embeddings $x_{\text{seq}},\;x_{\text{local}}$ and $x_{\text{pre}}$, are concatenated. This integrated feature vector is subsequently input into a Multi-Layer Perceptron (MLP) for classification. The objective of the MLP is to discern cell-penetrating peptides and to facilitate this, we use the cross-entropy loss function during the training phase.

## 3 Results

In this research, we present the model PractiCPP, which is tailored to address the challenges presented by realistic scenarios in cell-penetrating peptide (CPP) prediction, facilitating its practical deployment in real-world settings. Specifically, in many practical contexts, we have a small number of known positive CPP samples, while a large number of peptides have an undetermined CPP status (CPP or non-CPP). Therefore, our study focus on imbalanced binary classification, with a positive-to-negative sample ratio of 1:1000. However, prior efforts on CPP prediction have largely centered on balanced datasets, like the commonly used CPP924 dataset, which contains 462 CPPs and 462 non-CPPs. Thus, to validate the effectiveness of PractiCPP, we first benchmark it against state-of-the-art models on the balanced CPP924 dataset. We then exhibit the superiority of PractiCPP in tackling imbalanced CPP data.

### 3.1 Performance comparison on balanced data

Table 1 shows the result comparison between the proposed PractiCPP and seven top-performing baselines, i.e. CellPPD-1, CellPPD-2, CellPPD-3 (Gautam *et al.* 2013), StackCPPred (Fu *et al.* 2020), SkipCPP-Pred (Wei *et al.* 2017a), TargetCPP (Arif *et al.* 2020), and CPPred-RF (Wei *et al.* 2017b) on dataset CPP924. For the evaluation of balanced data, we utilize accuracy, sensitivity, specificity, and MCC as metrics. To ensure a fair comparison, we train the model on CPP924 and present the results of PractiCPP’s 10-fold cross-validation. In the training phase of PractiCPP, hard negative sampling is unnecessary given that CPP924 is a balanced dataset. Thus, we directly train the PractiCPP base model, PractiCPP${\;}_{\text{base}}$.

**Table 1.** Performance comparison on dataset CPP924 (10-fold cross validation).^a^

Method	**Acc** (%)	**Sn** (%)	**Sp** (%)	**MCC** (%)
CellPPD-1	90.70	90.90	90.50	81.60
CellPPD-2	87.00	83.30	90.70	74.50
CellPPD-3	83.70	78.10	89.20	68.00
SkipCPP-Pred	90.60	88.50	92.60	81.20
CPPred-RF	91.60	90.50	92.60	83.10
TargetCPP	93.54	93.41	93.68	87.10
StackCPPred	94.50	94.20	94.80	89.00
PractiCPP${\;}_{\text{base}}$	**95.65**	**94.29**	**97.06**	**91.34**

a The evaluation metrics for balanced data are accuracy (Acc), sensitivity (Sn), specificity (Sp), and MCC. The best results are highlighted in bold.

From Table 1, we observe that PractiCPP consistently outperforms seven baselines in terms of above four metrics, achieving an accuracy of 95.65%, sensitivity of 94.29%, specificity of 97.06% and MCC of 91.34%. Compared to the best baseline (StackCPPred), our model shows the relative improvements of 1.22% in accuracy, 2.38% in specificity and 2.63% in MCC while achieving a comparable sensitivity.

### 3.2 Performance comparison on imbalanced data

To evaluate the performance of different methods on imbalanced CPP classification, we split out an independent test set where the ratio of positive to negative samples maintains 1:1000, and report models’ results on this test set as in Tables 2 and 3. We benchmark against three methods: SiameseCPP (Zhang *et al.* 2023), BChemRF-CPPred (de Oliveira *et al.* 2021), and ML-CPP2 (Manavalan and Patra 2022). We train SiameseCPP on our imbalanced dataset. For BChemRF-CPPred and ML-CPP2, we use their released web servers for CPP prediction. All these three baselines are state-of-the-art in CPP prediction tasks and show great capability in CPP prediction on balanced data, such as CPP924 dataset, but lack a specific design to handle highly imbalanced data. In addition, to emphasize the necessity of hard negative sampling in our proposed model PractiCPP, we also compare PractiCPP with PractiCPP${\;}_{\text{base}}$, the variant includes only the feature extraction and prediction module. During the PractiCPP${\;}_{\text{base}}$ training process, uniform sampling, rather than the hard negative sampling, is leveraged to form the negative training set. In Tables 2 and 3, in addition to AUPR, we report precision, F1 score, and FP per correct at recall of 0.6 and 0.7, respectively. We observe that:

**Table 2.** Results on the 1:1000 independent test set.^a^

Method	AUPR	Rec	Prec	F1	FP/C
PractiCPP	**0.6400**	0.6	**0.8056**	**0.6864**	**0.2414**
PractiCPP${\;}_{\text{base}}$	0.5977	0.6	0.3841	0.4677	1.6034
SiameseCPP	0.5645	0.6	0.1662	0.2601	5.0172
BChemRF-CPPred	0.1210	0.6	0.0030	0.0019	527.65
ML-CPP2	0.0110	0.6	0.0021	0.0730	921.379

a For a fair comparison, the decision thresholds of PractiCPP and baselines are adjusted to yield a recall (Rec) of 0.6. The evaluation metrics for imbalanced data are precision (Prec), F1 score (F1), and FP per correct (FP/C). The best results are highlighted in bold.

**Table 3.** Results on the 1:1000 independent test set.^a^

Method	AUPR	Rec	Prec	F1	FP/C
PractiCPP	**0.6400**	0.7	**0.2048**	**0.317**	**3.8824**
PractiCPP${\;}_{\text{base}}$	0.5977	0.7	0.1193	0.2039	7.3823
SiameseCPP	0.5645	0.7	0.0961	0.1692	9.3971
BChemRF-CPPred	0.1210	0.7	0.0032	0.0016	616.39
ML-CPP2	0.0110	0.7	0.0018	0.0520	1120.529

a The decision thresholds are adjusted to achieve a recall of 0.7. The best results are highlighted in bold.

**Figure 2.**
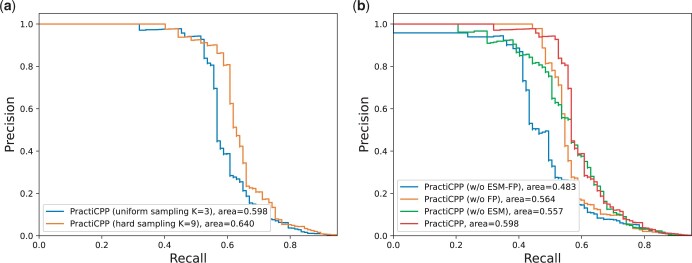
(a) Precision–recall curves of PractiCPP under uniform sampling (*K* = 3) and hard sampling (*K* = 9) on the 1:1000 test set. (b) Precision–recall curves of PractiCPP and its three variants, with K set to 3 (without using hard negative sampling).

- In this experimental setting with a positive-to-negative ratio of 1:1000, PractiCPP yields the best performance among all the baselines under AUPR and other metrics (precision, F1 score and FP per correct) at recall of 0.6 and 0.7, exhibiting the superiority of our method. Specifically, in terms of AUPR, PractiCPP outperforms the best baseline (SiameseCPP) by a large margin, the relative improvement reaching 7.08%. At a recall of 0.6, PractiCPP achieves a precision of 0.8056, which is a substantial improvement over SiameseCPP’s 0.1662, while at a recall of 0.7, the performance gap between PractiCPP and other methods is reduced. Nevertheless, PractiCPP’s precision (0.2048) is still more than twice that of SiameseCPP (0.0961). For BChemRF-CPPred and ML-CPP2 which are trained on the limited-scale dataset and do not specifically adjust for imbalanced samples during model training, their accuracies (0.0032 and 0.0018, respectively) are superior to random guessing (0.001) on the 1:1000 imbalanced dataset, but remain insufficient for addressing real-world tasks.
- PractiCPP consistently performs better than the variant PractiCPP${\;}_{\text{base}}$, exhibiting a 7.08% improvement over PractiCPP${\;}_{\text{base}}$ in terms of AUPR, revealing that the hard negative sampling plays a critical role in imbalanced CPP classification. The precision–recall curves of these two methods are also drawn in Fig. 2a. From Fig. 2a, we observe that for recall values below 0.5 and above 0.8, the precision metrics of the two methods exhibit slight difference, but in the recall range of 0.5–0.8, PractiCPP’s precision notably surpasses that of PractiCPP${\;}_{\text{base}}$. This insight supports our model’s real-world deployments, as it can benefit from achieving good precision at a relatively high recall.

### 3.3 Ablation study

In the aforementioned two sections, we already study the impact of the hard negative sampling technique in PractiCPP, and exhibit its great contribution to the model’s overall performance. To investigate how much other components of PractiCPP influence the final performance, we conduct an ablation study in this section. We use PractiCPP with uniform sampling (*K *=* *3) as the baseline and compare it with three variants.

- PractiCPP (w/o ESM): PractiCPP without embeddings generated from pretrained model ESM-2.
- PractiCPP (w/o FP): PractiCPP without local feature embeddings derived from peptide Morgan fingerprints.
- PractiCPP (w/o ESM-FP): PractiCPP without both ESM-2 pretrained embeddings and local feature embeddings.

**Figure 3.**
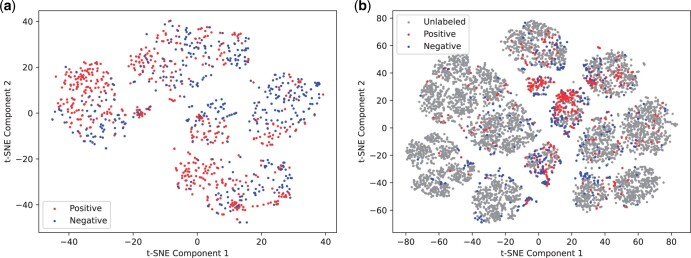
The t-SNE visualization of peptides’ Morgan fingerprints. (a) The t-SNE plot of Morgan fingerprints from CPPs (positives) and non-CPPs (negatives). (b) The t-SNE plot of Morgan fingerprints from CPPs, non-CPPs, and unlabeled peptides.

The precision–recall curves of above four methods are drawn in Fig. 2b. We observe that PractiCPP (w/o ESM-FP) performs the worst, only achieving 0.483 in terms of AUPR. In addition, the relative improvements of PractiCPP over PractiCPP (w/o FP) and PractiCPP (w/o ESM) are 6.03% and 7.36% respectively, indicating that both ESM pretrained model and Morgan fingerprints can be beneficial in CPP prediction, and the pretrained embeddings contribute more to the model performance than Morgan fingerprints information. To further explain why we add Morgan fingerprints to our model, we use t-SNE to visualize the Morgan fingerprint distributions across CPPs, non-CPPs and unlabeled peptides as in Fig. 3. Here, non-CPPs are the 462 negative instances in dataset CPP924. CPPs and unlabeled peptides are from the 1:1000 dataset in our experiments. To provide a clear visualization without overwhelming the figure, we have randomly selected 6490 unlabeled peptides as a representative subset of the entire unlabeled distribution. Figure 3a and b presents the clustering of CPPs, non-CPPs, and unlabeled peptides, illustrating their shared chemical properties. The distributions of CPPs and non-CPPs are similar, yet exhibit slight shifts, which could be instrumental in their classification. Unlabeled peptides display a wider clustering, where CPPs and non-CPPs sourced from CPP924 center in several certain clusters, which highlights the significance of Morgan fingerprint in distinguishing CPPs. More detailed analyses can be found in Supplementary Note S3.

### 3.4 Embedding visualization

In this section, we visualize the embeddings of PractiCPP to highlight the significance of learning on highly imbalanced positive-unlabeled datasets. We use two trained models for deriving embeddings: (i) PractiCPP trained on the balanced positive-negative CPP924 dataset, (ii) PractiCPP trained on our 1:1000 positive-unlabeled dataset. Their embeddings of CPPs, non-CPPs and unlabeled peptides are generated from the penultimate fully connected layer of PractiCPP’s MLP. These are then projected into a 2D space using t-SNE.

**Figure 4.**
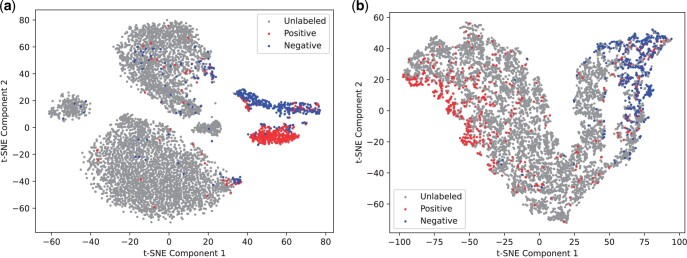
The t-SNE visualization of PractiCPP generated embeddings for CPPs, non-CPPs, and unlabeled peptides. In (a), PractiCPP is trained on the 1:1000 imbalanced dataset. In (b), PractiCPP is trained on the balanced dataset CPP924.

As shown in Fig. 4a, PractiCPP, when trained on imbalanced data, clearly separates three groups: CPPs, non-CPPs, and peptides without labels, with few instances where they overlap. Conversely, when trained on the balanced dataset (as in Fig. 4b), although CPPs and non-CPPs are separated, they fail to distinctly separate from unlabeled peptides. This suggests that models trained on balanced data might be impractical for guiding large-scale peptide selection for wet-lab experiments in CPP identification. In contrast, training with imbalanced data has the potential to empower real-world applications.

### 3.5 Motif visualization

**Figure 5.**
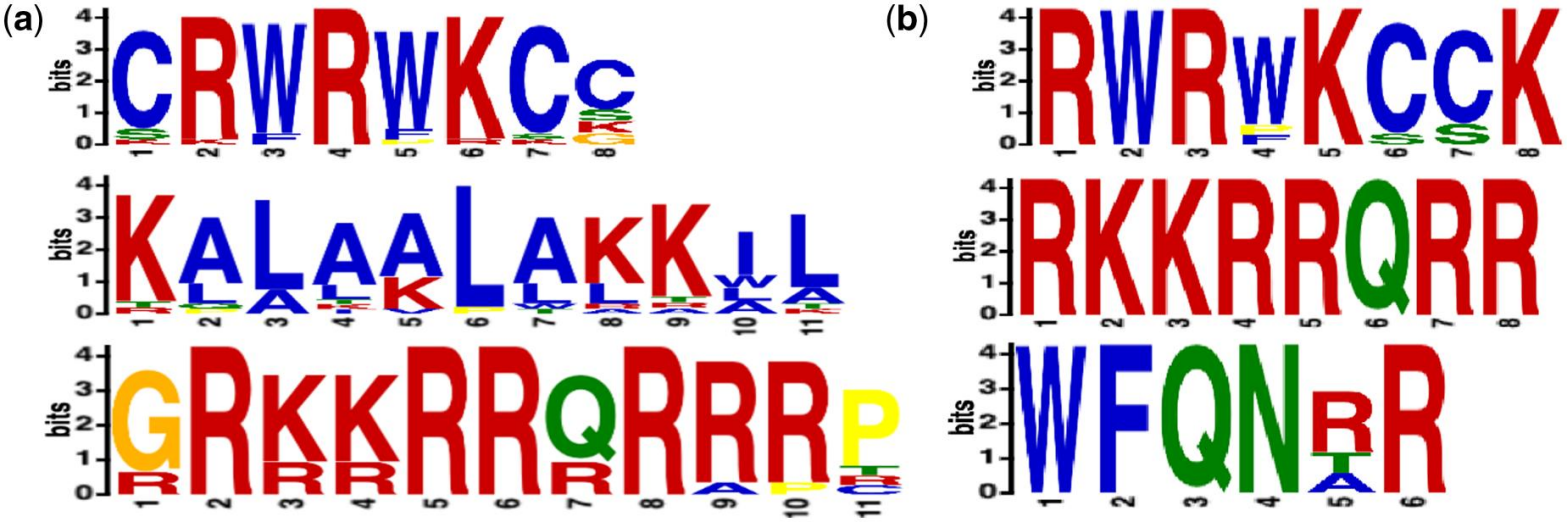
Sequence motifs captured by PractiCPP. (a) Motifs captured from real-world CPPs. The enriched ‘R’ and ‘K’ are reported to be classical components of functional CPPs. (b) Motifs captured from CPPs predicted by PractiCPP. In addition to the ‘R’ and ‘K’ components, the ‘F’ and ‘Q’ are also suggested by PractiCPP.

As shown in the previous section, the feature embedding capability of PractiCPP has effectively transformed the original, inseparable features into separable, high-dimensional ones, enabling accurate discrimination between positive and negative samples. In this section, we aim to dive deeper into visualizing and analyzing the sequence features captured by the model, including both real-world and predicted samples. More specifically, we first use the MEME suite (Bailey *et al.* 2015) to calculate sequence motifs of 649 real CPPs. These sequences are then ranked based on the statistical significance provided by MEME, and top motifs are selected (Fig. 5a). This process reveals the sequence characteristics present in real-world data with existing labels. As a result, Arginine(R) and Lysine(K) are prevalent in the initial motifs, indicating their role as typical components in cationic cell-penetrating peptides (Schmidt *et al.* 2010). Pei *et al.*’s research also supports this, suggesting that functional cell-penetrating peptides require at least two arginines (Dougherty *et al.* 2019). Tryptophan (W) and Leucine (L) are also identified as the significant elements of hydrophobic peptides, i.e. CorTS1 and RW9 (Chan *et al.* 2006, Wang *et al.* 2017). While the 649 cell-penetrating peptides exhibit these typical features, we broaden our analysis by applying PractiCPP to a wider range of unlabeled natural peptides and virtually design peptides to uncover new characteristics. We process all samples from the independent test set through PractiCPP, identifying sequences deemed as positive samples (at a recall of 0.65). Using MEME, we calculate motifs for these sequences and rank their features based on statistical significance (as shown in Fig. 5b). This analysis reveals that the sequences identified by the model as cell-penetrating peptides not only confirm the classical components (R, W, K) but also highlight three novel components: Glutamine(Q), Asparagine(N), and Phenylalanine(F) (though Q being less prominent in the original data). Although further validation is needed for the role of Glutamine and Asparagine, Phenylalanine is known to enhance cell penetration and also has significant associations with passive cell permeability (Sayers *et al.* 2014). These analyses demonstrate the potential of our model to contribute new insights to the field of cell-penetrating peptides in the real world.

## 4 Discussion

In this paper, we introduce PractiCPP, a deep learning framework specifically designed to address the challenges posed by imbalanced binary classification in CPP prediction. Our model achieves state-of-the-art performance on both balanced and imbalanced datasets, underscoring the efficacy of our approach. The hard negative sampling technique plays a pivotal role in enhancing the model’s performance, as it compels the model to concentrate on challenging negative samples, refining its decision boundaries, and augmenting its overall performance. The visualization of the model’s embeddings demonstrates PractiCPP’s ability to distinguish between CPPs, non-CPPs, and unlabeled peptides when trained on imbalanced data. In conclusion, PractiCPP offers a promising solution for the prediction of CPPs in real-world scenarios, where only a limited number of known positive CPP samples are available. The model’s ability to handle imbalanced data and its state-of-the-art performance on both balanced and imbalanced datasets demonstrate its potential for practical deployment in drug delivery research and development. We believe that the success of PractiCPP in predicting CPPs could inspire its application to other related problems in computational biology and chemistry, such as protein–protein interaction prediction, drug–target interaction prediction, and protein structure prediction. By adapting the framework to these tasks, we may be able to develop novel methods to tackle these challenges more effectively.

## Supplementary Material

btae058_Supplementary_Data

## Data Availability

The PractiCPP algorithm and the balanced data CPP924 used in this article are available on Figshare and can be accessed via link https://doi.org/10.6084/m9.figshare.25053878.v1. The 1:1000 imbalanced data constructed in this article will be shared on reasonable request to the corresponding author.
