# Supplementary material for: PractiCPP: a deep learning approach tailored for extremely imbalanced datasets in cell-penetrating peptide prediction
Source: Bioinformatics. 2024 Feb 1;40(2):btae058. doi: 10.1093/bioinformatics/btae058 (PMC11212486; doi:10.1093/bioinformatics/btae058)
Supplement: btae058_Supplementary_Data [file btae058_supplementary_data.pdf]

# Supplementary Notes for "PractiCPP: A Deep Learning Approach Tailored for Extremely Imbalanced Datasets in Cell-Penetrating Peptide Prediction"

Kexin Shi[1,2,†] Yuanpeng Xiong[1,†] Yu Wang[1] Yifan Deng[1] Wenjia Wang[3]
Bingyi Jing[4,*]
Xin Gao[1,5,6,**]
[1]Syneron Technology, Guangzhou 510000, China
[2]Information Hub, The Hong Kong University of Science and Technology, Kowloon 999077, Hong Kong
[3]Information Hub, The Hong Kong University of Science and Technology, Guangzhou 511455, China
[4]Department of Statistics and Data Science, Southern University of Science and Technology, Shenzhen 518000, China
[5]Computer Science Program, Computer, Electrical and Mathematical Science and Engineering Division, King Abdullah University of Science and Technology (KAUST), Thuwal 23955, Saudi Arabia
[6] Computational Bioscience Research Center, King Abdullah University of Science and Technology (KAUST), Thuwal 23955, Saudi Arabia

## S1 Evaluation metrics

For experiments where the ratio of positives to negatives is 1:1, we adopt four metrics: accuracy, sensitivity (or recall), specificity and the Matthews correlation coefficient (MCC) to evaluate models. Note that sensitivity is crucial for evaluating how well the model captures positive occurrences, and specificity indicates the model's ability to discern true negative instances accurately.

In contrast, for experiments where the ratio of positives to negatives is 1:1000, some evaluation metrics like accuracy can be misleading [1], thus we use metrics that account for this data imbalance: recall (or sensitivity), precision, F1 score and FP per correct. In addition, AUPR is used to capture the trade-off between precision and recall. Note that precision is crucial to monitor how accurately the model predicts the rate of positive cases because given the large number of negative instances, even a small percentage of misclassifications can translate into a large absolute number of false positives.

[*]Corresponding authors. Email: jingby@sustech.edu.cn, xin.gao@kaust.edu.sa
†These authors contribute equally.

The evaluation metrics used in the paper are computed as follows:

$$\text{accuracy} = \frac{\text{TP} + \text{TN}}{\text{TP} + \text{TN} + \text{FP} + \text{FN}},$$

$$\text{sensitivity} = \frac{\text{TP}}{\text{TP} + \text{FN}},$$

$$\text{specificity} = \frac{\text{TN}}{\text{TN} + \text{FP}},$$

$$\text{MCC} = \frac{\text{TP} \times \text{TN} - \text{FP} \times \text{FN}}{\sqrt{(\text{TP} + \text{FP})(\text{TP} + \text{FN})(\text{TN} + \text{FP})(\text{TN} + \text{FN}}},$$

$$\text{precision} = \frac{\text{TP}}{\text{TP} + \text{FP}},$$

$$\text{F1 score} = 2 \times \frac{\text{precision} \times \text{recall}}{\text{precision} + \text{recall}},$$

$$\text{FP per correct} = \frac{\text{FP}}{\text{TP}},$$

where TP, TN, FP, and FN denote true positives, true negatives, false positives, and false negatives respectively.

# S2 Hardness study

Towards hard negative sampling in PractiCPP, we conduct a hardness study to take a closer look at how the hard negatives contribute to the model's performance improvements. Notably, a larger $K$ generally indicates harder negatives selected. Specifically, a larger $K$ means a larger negative candidate pool from the overall negative set, raising the likelihood of including truly hard negatives. Thus, the top $3 \times |\text{positives in batch}|$ negatives scored by the model are more likely to be truly challenging negatives. Note that at $K = 3$, the hard negative sampling strategy degrades to a uniform sampling approach.

In Figure S1a and Figure S1b, we show AUPR and AUROC values under various $K$ setting ($K$ in $(3, 9, 15, 21, 30)$). In our evaluations, the optimal performance for PractiCPP is observed at $K = 9$, where the AUPR reaches a peak value of 0.6400 and the AUROC achieves 0.9600. Then with $K$ increasing, AUPR drops to around 0.6200, and AUROC even drops from 0.96 to 0.93 at $K = 30$. It is not the case that harder negatives yield better results. In fact, selecting an appropriate $K$ is critical. One potential explanation for this phenomenon is that excessively challenging negatives could include potential positive samples, thus misleading the model training.

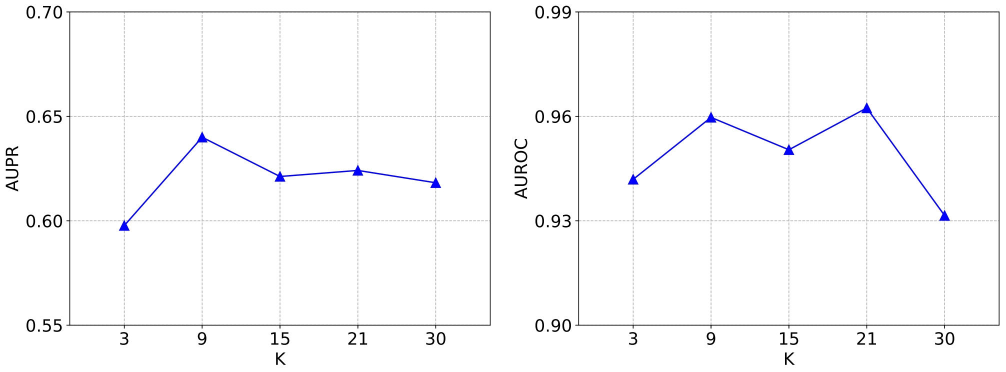


(a) (b)

Figure S1: (a) The influence of the selected negatives' hardness level on the AUPR performance of PractiCPP. (b) The influence of the selected negatives' hardness level on the AUROC performance of PractiCPP. A larger K indicates harder negative selection.

# S3 The importance of Mogran fingerprint

The observations from Fig. 3 in the manuscript are as follows:

- Peptides are grouped into distinct clusters. This could be due to specific chemical functional groups or substructures within the peptides, causing them to have similar fingerprint representations and thus cluster together.

- The fingerprint distributions of CPPs and non-CPPs are close but exhibit a shift (in Fig. 3a). This may be because the non-CPPs sourced from CPP924 are peptides that resemble CPPs in their attributes but demonstrate low cell-penetrating capability in wet-lab experiments. The shift in their fingerprint distributions may provide insights into classifying CPPs from non-CPPs.

- Unlabeled peptides display a wider clustering, where CPPs and non-CPPs sourced from CPP924 center in several certain clusters (in Fig. 3b). This suggests the greater structural and attribute diversity of extensive natural peptides, and Morgan fingerprint information is beneficial in distinguishing CPPs from the vast array of unlabeled peptides.

From the perspective of biology, in the process of peptide penetrating cellular membranes, various mechanisms are typically involved, such as passive penetration, translocation, and endocytosis [2]. Following the entry of peptides into the cell membrane via endocytosis, an important subsequent process might also occur, namely endosomal release [3]. The occurrence and efficiency of these processes are largely dependent on the specific functional groups present in the peptides. For instance, charged amino acids may engage in charge-charge interactions with glycoproteins on the surface of the cellular membrane [4], facilitating subsequent membrane penetration processes, while hydrophobic groups aid in the penetration of the hydrophobic cell membrane. Overall, the presence of specific functional groups in peptides is a critical factor in assessing their ability to penetrate cell membranes. The Morgan fingerprint, as a characteristic widely used in molecular description,

inherently includes detailed information about functional groups and is thus extensively employed in predicting the properties of molecules [5].

## References

[1] Yuanpeng Xiong, Xuan He, Dan Zhao, Tingzhong Tian, Lixiang Hong, Tao Jiang, and Jianyang Zeng. Modeling multi-species rna modification through multi-task curriculum learning. *Nucleic acids research*, 49(7):3719–3734, 2021.

[2] Patrick G Dougherty, Ashweta Sahni, and Dehua Pei. Understanding cell penetration of cyclic peptides. *Chemical Reviews*, 119(17):10241–10287, 2019.

[3] Alfredo Erazo-Oliveras, Nandhini Muthukrishnan, Ryan Baker, Ting-Yi Wang, and Jean-Philippe Pellois. Improving the endosomal escape of cell-penetrating peptides and their cargos: strategies and challenges. *Pharmaceuticals*, 5(11):1177–1209, 2012.

[4] Isabel D Alves, Nicole Goasdoué, Isabelle Correia, Soline Aubry, Cécile Galanth, Sandrine Sagan, Solange Lavielle, and Gérard Chassaing. Membrane interaction and perturbation mechanisms induced by two cationic cell penetrating peptides with distinct charge distribution. *Biochimica et Biophysica Acta (BBA)-General Subjects*, 1780(7-8):948–959, 2008.

[5] Vinod Kumar, Piyush Agrawal, Rajesh Kumar, Sherry Bhalla, Salman Sadullah Usmani, Grish C Varshney, and Gajendra PS Raghava. Prediction of cell-penetrating potential of modified peptides containing natural and chemically modified residues. *Frontiers in microbiology*, 9:725, 2018.
